# Uncovering the flip side of immune checkpoint inhibitors: a comprehensive review of immune-related adverse events and predictive biomarkers

**DOI:** 10.7150/ijbs.89376

**Published:** 2024-01-01

**Authors:** Yan-Dong Miao, Wu-Xia Quan, Xiao-Long Tang, Wei-Wei Shi, Qing Li, Rui Jian Li, Jiang-Tao Wang, Jian Gan, Xin Dong, Liang Hao, Wen-Yu Luan, Fang Zhang

**Affiliations:** 1 Cancer Center, Yantai Affiliated Hospital of Binzhou Medical University, The 2 nd Medical College of Binzhou Medical University, Yantai 264100, China.; 2 Yantai Affiliated Hospital of Binzhou Medical University, The 2 nd Medical College of Binzhou Medical University, Yantai 264100, China.; 3 The First Clinical Medical College, Lanzhou University, Lanzhou 730000, China.; 4 Department of Thyroid and Breast Surgery, Yantai Affiliated Hospital of Binzhou Medical University, The 2 nd Medical College of Binzhou Medical University, Yantai 264100, China.; 5 Department of Gastroenterology, Yantai Affiliated Hospital of Binzhou Medical University, The 2 nd Medical College of Binzhou Medical University, Yantai 264100, China.

**Keywords:** Cancer, Immunotherapy, Immune Checkpoint Inhibitors, Immune-Related Adverse Effects, Markers

## Abstract

Immune checkpoint inhibitors (ICIs) have generated considerable excitement as a novel class of immunotherapeutic agents due to their remarkable efficacy in treating various types of cancer. However, the widespread use of ICIs has brought about a number of safety concerns, especially the development of immune-related adverse events (irAEs). These serious complications could result in treatment discontinuation and even life-threatening consequences, making it critical to identify high-risk groups and predictive markers of irAEs before initiating therapy. To this end, the current article examines several potential predictive markers of irAEs in important organs affected by ICIs. While retrospective studies have yielded some promising results, limitations such as small sample sizes, variable patient populations, and specific cancer types and ICIs studied make it difficult to generalize the findings. Therefore, prospective cohort studies and real-world investigations are needed to validate the potential of different biomarkers in predicting irAEs risk. Overall, identifying predictive markers of irAEs is a crucial step towards improving patient safety and enhancing the management of irAEs. With ongoing research efforts, it is hoped that more accurate and reliable biomarkers will be identified and incorporated into clinical practice to guide treatment decisions and prevent the development of irAEs in susceptible patients.

## Introduction

Normally, the body's immune system is a complex and powerful defense mechanism that can usually identify and eradicate tumor cells within the tumor microenvironment. However, the survival and growth of tumor cells are facilitated via their adoption of various strategies that allow them to evade the immune system's detection and attack. As a result, the immune system becomes suppressed and incapable of effectively eliminating the tumor cells, leading to their survival throughout the different stages of the anti-tumor immune response 1. The above trait of tumor cells is known as immune escape. In order to gain a deeper understanding of the complexity of multi-link and multi-step tumor immunity, the concept of the tumor-immune cycle was introduced by Chen, et al 2. Various types of tumors can interfere with the immune system's capability to effectively recognize and eliminate cancer cells by disrupting different stages of the cycle, thereby promoting immune tolerance and contributing to the development of tumors.

**Figure 1:**
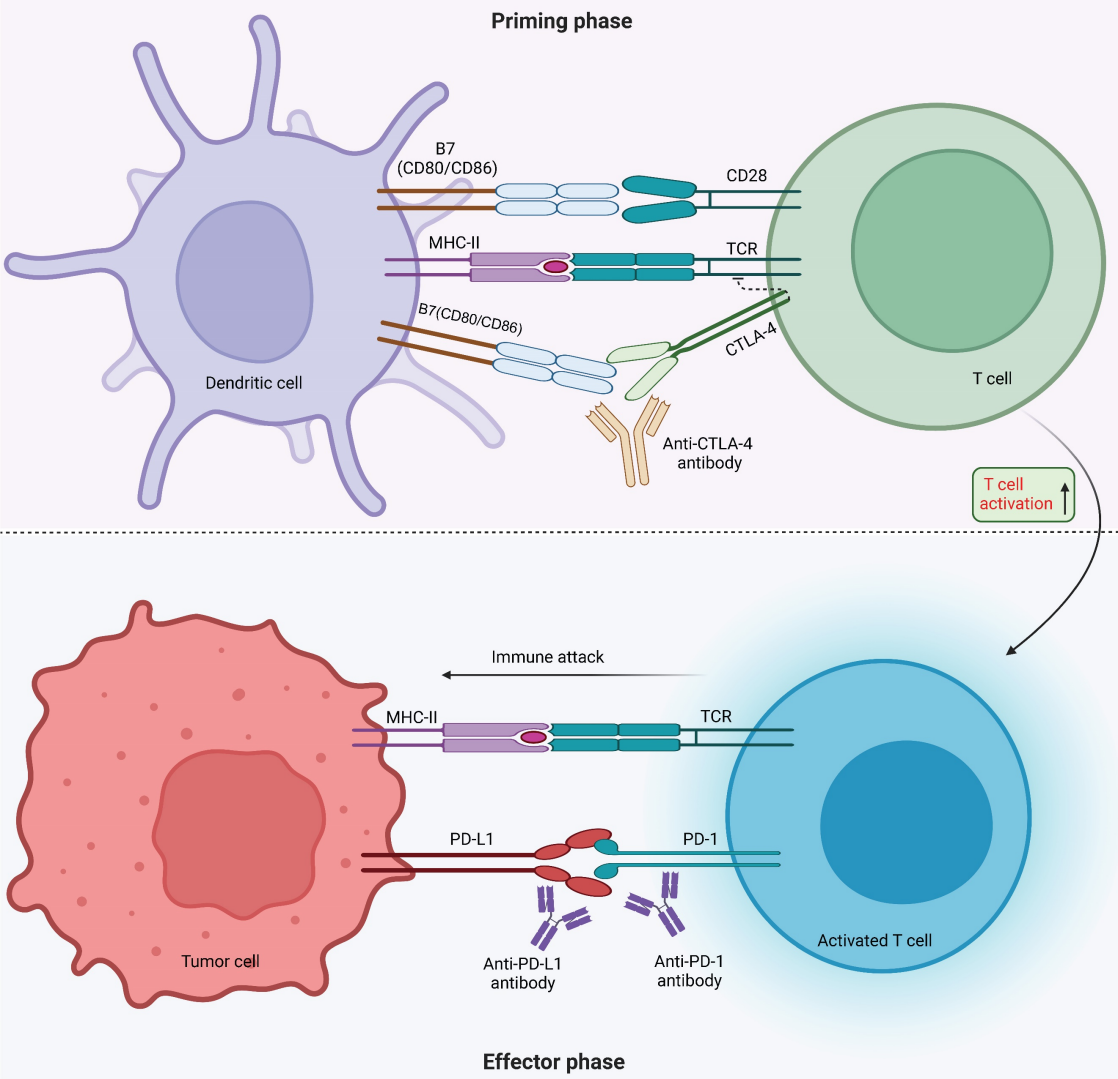
** Mechanism of immune checkpoint inhibitors.** Monoclonal antibodies targeting CTLA-4 and PD-1 receptors, as well as PD-L1, are ICIs that regulate T cell activation. During the priming phase, antigen presentation by MHC class II molecules on antigen-presenting cells triggers T cell activation by the T-cell receptor (TCR) recognizing antigen, followed by CD28 receptor binding with B7 (CD80 or CD86). The surface receptor of CTLA-4 on T cells inhibits T cell activation through competing with CD28 for CD80 or CD86 binding. The use of CTLA-4 inhibitor antibodies blocks the CTLA-4-CD80 or CTLA-4-CD86 binding, thus promoting T cell activation (indicated by a dashed line). During the effector phase, PD-1 expressed by T cells interacts with PD-L1 expressed by tumor and myeloid cells, promoting apoptosis of antigen-specific T cells while reducing regulatory T cell apoptosis. Under normal circumstances, this mechanism serves to protect against autoimmune disorders. However, cancer cells take advantage of it by increasing the expression of PD-L1, which helps them evade the immune system. To counteract this, inhibitors of PD-1 and PD-L1 can be used to block their interaction and promote activation of T-cells.

**Figure 2:**
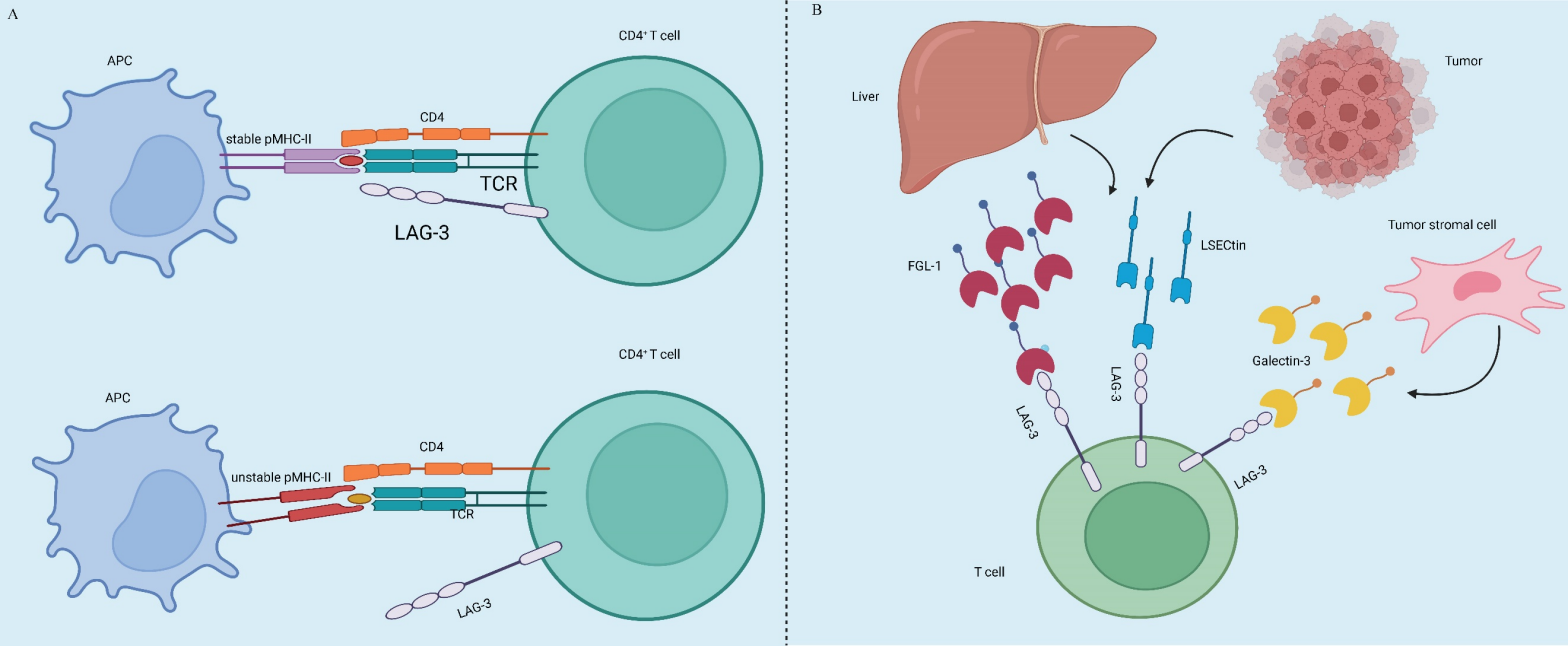
** LAG-3 expression and ligands. (A)** LAG-3 is not initially present on primary T cells but can be induced upon antigen stimulation on both CD4+ and CD8+ T cells. It is also expressed in a particular subset of CD4+ T cells with suppressive capabilities. LAG-3 can selectively bind to stable pMHCII7, distinguishing the conformation of pMHCII^11^. **(B)** Additionally, FGL1 protein is a significant functional ligand for LAG-3. Upregulation of FGL1 expression by tumor cells can regulate the inhibitory function of LAG-3, thereby affecting T cell immune activity 12. Furthermore, Galectin-3 and LSECtin can interact with the glycans on LAG-3 9, 13.

Tumor immunotherapy aims to manage and eliminate tumors by reinitiating and sustaining the tumor-immune cycle, thereby reinstating the body's typical anti-tumor immune response 3. It includes immune checkpoint inhibitors (ICIs), cell therapy, small molecule inhibitors and cancer vaccines. Among the many immunotherapeutic strategies, ICIs are widely used and have shown remarkable efficacy in managing various types of solid tumors, including but not limited to non-small cell lung cancer (NSCLC), melanoma, bladder cancer, kidney cancer, and prostate cancer, among others. By using antibodies against cytotoxic T lymphocyte-associated protein 4 (CTLA-4), and inhibiting programmed cell death 1 (PD-1) or its ligand 1 (PD-L1), ICIs improve the immune response against tumors 4. CTLA4, the initial immune-checkpoint receptor to undergo clinical targeting, is exclusively expressed on T cells, where its primary role is to modulate the magnitude of early T cell activation 5. CTLA-4 molecules exhibit a stronger binding affinity to CD80 and CD86 compared to CD28, and they function as competitive inhibitors of CD28 in antigen-presenting cells (APCs) 6. Cancer cells frequently co-opt the PD-1 signaling pathway as a means to evade immune surveillance. Moreover, apart from inhibiting specific early activation pathways of T cells, PD-1 directly impairs antigen recognition by disrupting the trimolecular interaction involving TCR-pMHC-CD8 (Figure 1). In addition to these immune checkpoints, there are many immune checkpoints that exhibit immunotherapeutic properties, including V structural domain Ig suppressor of T cell activation (VISTA, also known as VSIR), TIM-3 (also known as HAVCR2), lymphocyte-activated gene-3 (LAG-3, also known as CD223), T cell Ig and ITIM domain (TIGIT, WUCAM, Vstm3, VSIG9), Signal regulatory proteins (SIRP), BTLA (CD272), Siglec-7, LILRB, et al 7, 8. LAG-3 is currently considered a prime target after PD-1/L1 (Figure 2). Several clinical trials are validating the effectiveness of LAG-3-targeted therapy 9, and Opdualag, the initial fixed-dose combination of Relatlimab, a LAG-3 inhibitor, and nivolumab, a PD-1 inhibitor, has been authorized by the Food and Drug Administration (FDA) on March 19, 2022, for treating unresectable or metastatic melanoma in patients aged 12 or above. This is the first combination regimen of a LAG-3 inhibitor and a PD-1 inhibitor to receive formal marketing approval, based on the results of RELATIVITY-047 phase II/III trial 10. Multiple ICIs can significantly improve overall survival in patients with various cancers and have been approved for clinical use by the FDA and National Medical Products Administration (NMPA) (Table 1).

Immunotherapy kills tumors by improving the body's own immune function, but an over-activated immune system can also damage normal cells due to complex mechanisms, resulting in uncomfortable symptoms in the relevant organs, called immunotherapy-related adverse events (irAEs). The use of ICIs for cancer treatment has resulted in a sharp rise in the cumulative annual incidence of irAEs, with almost 13,000 cases reported in 2018 14. ICIs exhibit a distinct spectrum of adverse effects compared to conventional chemotherapy and other biological treatments. The majority of these adverse events stem from an overactive immune response targeting healthy tissues, affecting nearly every organ system 4, 15. Notably, more than two-thirds of documented cases are irAEs, with three specific drugs (ipilimumab, nivolumab, and pembrolizumab) accounting for nearly 60% of these incidents 14. The severity of these toxicities varies from mild to life-threatening, creating a clinical dilemma regarding whether to discontinue treatment or manage the condition through frequent hospitalization and immunosuppressive therapy 16-18. In this review, we mainly discuss the irAEs and predictive biomarkers.

## Immunotherapy-related adverse events

**Figure 3:**
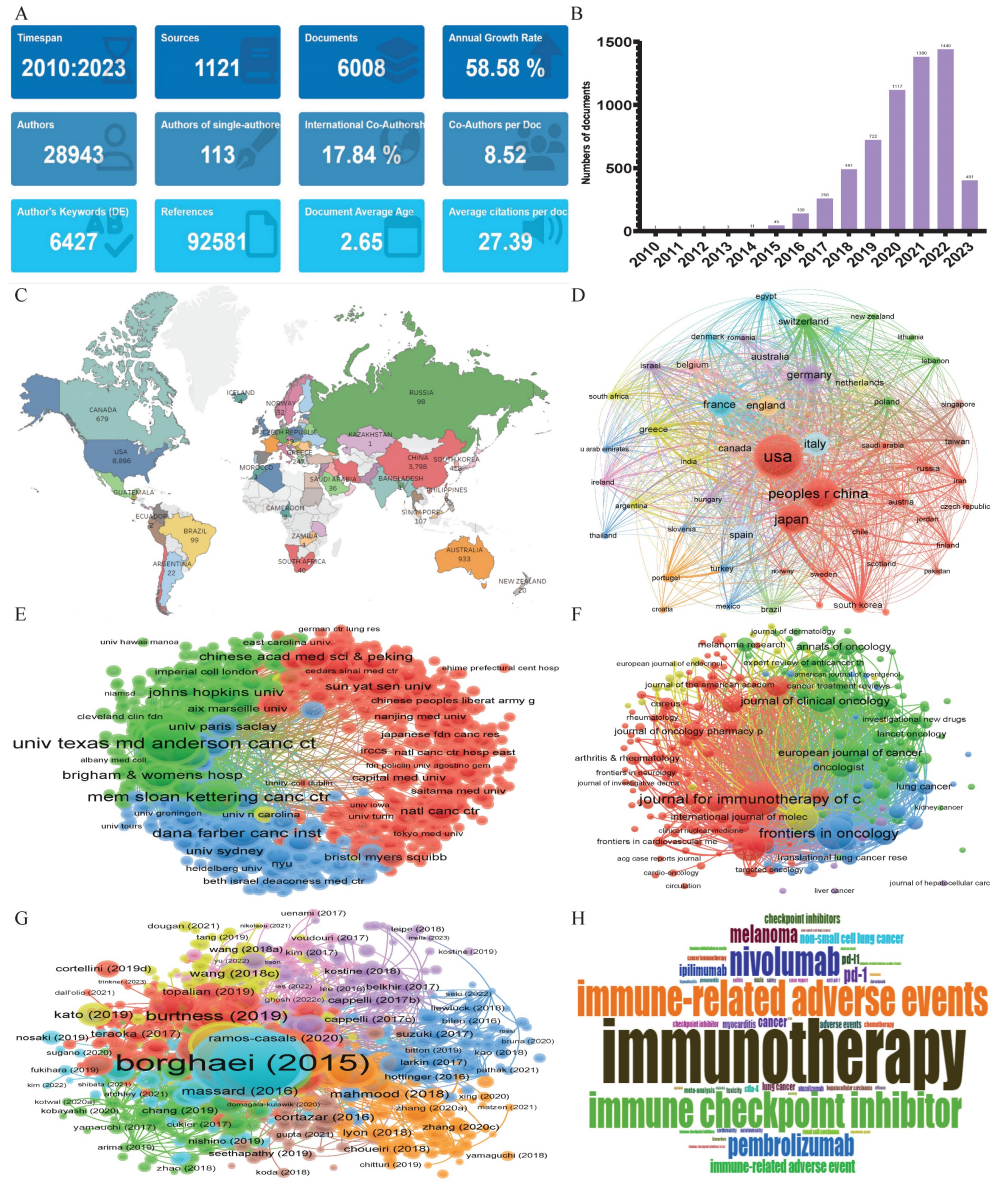
** A bibliometric analysis of adverse events related to immune checkpoint inhibitors.** (A) The overview of adverse events related to immune checkpoint inhibitors in WOSCC by 'Bibiometrix' package. (B) Analysis of annual publication trends in the field of adverse events related to immune checkpoint inhibitors. (C) World map of Conutries's scientific production. (D) A geographical distribution of scholarly papers originating from various nations, elucidated through bibliographic coupling analysis facilitated by VOSviewer (version 1.6.18). Every circle symbolizes a nation, with the circle's magnitude corresponding to the volume of scholarly production from that nation. The connecting lines imply inter-country collaborations; the thicker the line, the more intimate the cooperation. Various hues signify distinct clusters. € An elucidation of the distribution of scholarly output emanating from different institutions, facilitated via bibliographic coupling analysis using VOSviewer (version 1.6.18). Every circle represents an institution, and the circle's magnitude embodies the volume of scholarly output from that institution. Interconnecting lines suggest institutional collaborations; the broader the line, the more intimate the association. Diverse shades indicate distinct clusters. (F) A VOSviewer visualization chart of bibliographic coupling sources. Each circle embodies a journal, with the circle's size reflecting the volume of publications in that particular journal as per the bibliographic coupling analysis. The greater the circle, the more voluminous the publications. Lines connecting the circles signify inter-journal relationships, and differently hued connection networks suggest cooperative clusters between distinct journals. Diverse shades represent different clusters 22. (G) A VOSviewer visualization diagram of cited manuscripts. Each circle represents a manuscript, with the circle's size mirroring the citation count in the citation analysis. The greater the circle, the more citations. Interconnecting lines illustrate relationships between manuscripts, and distinctively hued connection networks indicate collaborative clusters between different documents. Various colors symbolize different clusters. (H) A word cloud representing the frequency of authors' keywords within the realm of unfavorable outcomes linked to immune checkpoint inhibitors. The more prominent the font, the greater its prevalence.

As shown in Figure 3, the bibliometric method was used to search the Web of Science core collection (WOSCC) literature database- Science Citation Index-Expanded (SCI-Expanded), based on the subject terms of “adverse event*” and “immune checkpoint inhibitor*”. 6008 English articles and reviews were published from January 2010 to April 2023 with an annual growth rate of 58.58%, particularly after 2019, signaling a rapid escalation in this field of research (Figure 3A, B).The most frequent countries' Scientific Production were the United States of America (8896), followed by China (3798) and Japan (3194), Moreover, the nations with the highest volume of publications in this field were the United States of America (1,886 articles, accounting for 31.39% of the total), China (1,115 articles, representing 18.56%), and Japan (780 articles, 12.98%), securing the top three positions respectively (Figure 3C, D). Between January 2010 and April 2023, the leading institutions in terms of the volume of global publications in the field of “adverse events related to immune checkpoint inhibitors” are illustrated in Figure 3E. “The University of Texas MD Anderson Cancer Center” and “Harvard Medical School” held the first two places, with a total of 613 and 303 publications, respectively. “The Memorial Sloan Kettering Cancer Center”, having published 287 articles, occupied the third position. The “Journal for Immunotherapy of Cancer”, with 259 publications, was the periodical that carried the most literature on the subject. “Frontiers in Oncology”, publishing 226 articles, and “Cancers”, with 175 publications, took the second and third positions respectively (Figure 3F). Figure 3G provides a schematic representation of the literature's co-citation network. The most frequently cited work was Borghaei H, 2015^19^, with 6,497 citations, followed by Brahmer J, 2015^20^ (4,676 citations), and Robert C, 2015 21 (3,896 citations) ranking second and third, respectively. An analysis based on keywords can reveal trends in theme evolution and research hotspots within a particular field over a given period. As shown in Figure 3H, the five most frequently occurring keywords were: “immunotherapy, immune checkpoint inhibitors, immune-related adverse events, immune checkpoint inhibitor, and nivolumab”.

**Figure 4:**
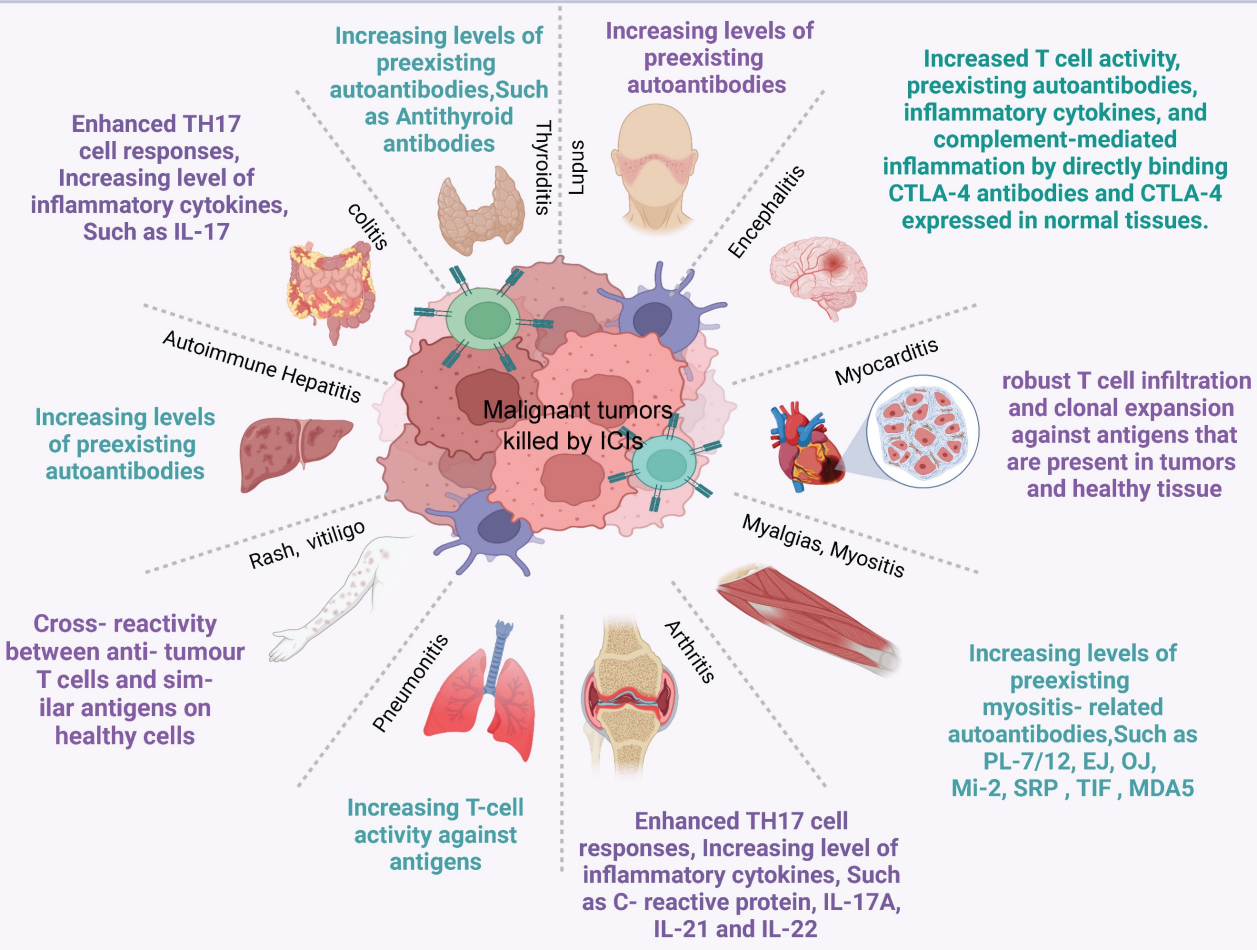
** Common immune-related adverse events and possible mechanisms.** ICI therapy may result in adverse events in any organ. This diagram shows the most common irAEs encountered by clinicians in patients treated with ICIs and their possible mechanisms.

irAEs can manifest in various organs, with the skin, gastrointestinal tract, kidney, endocrine gland, liver, and other organs being the most commonly affected (Figure 4, Figure S1). Also, potential irAEs involve a wide range of organs and require multidisciplinary and collaborative management by physicians from various disciplines in the clinical setting. When ICIs are used in tumor treatment, predicting the risk of irAEs before drug administration can effectively help identify high-risk groups, enabling enhanced surveillance, early intervention, and, in some cases, irAE prevention. It is particularly important to discover predictive markers for irAEs. For these reasons, this article also reviews the predictive markers of irAEs in several important organs caused by ICIs. Common irAEs and potential biomarkers for various irAEs are summarized in Table 2.

### Dermatologic-related Toxicity

Among the adverse events related to ICIs, dermatological toxicity is frequently observed, particularly in patients receiving CTLA-4 or PD-1/PD-L1 blockade 23. Incidences of dermatological toxicity (of all grades) have been reported in around 30-40% of patients taking PD-1/PD-L1 inhibitors and in about 50% ipilimumab treated patients 24. Dermatologic-related toxicity appears earlier compared to other irAEs, with cases being observed around one month after the commencement of anti-CTLA-4 treatment and approximately two months after starting PD-1/PD-L1 inhibitors 25, 26. The main clinical manifestations of dermatologic -related toxicity are macular papules, pruritus and vitiligo 4, 14, 27, 28. Other types include lichenoid dermatitis, psoriasis, autoimmune skin diseases (e.g., herpetiform aspergillosis, dermatomyositis, pemphigus), acne-like rash, and vascular disease-like changes 4, 14, 27.

A network meta-analysis of ICIs for 14 randomized clinical trials (RCTs) including 9572 advanced NSCLC patients demonstrated that combination of two ICIs or combination with chemotherapy drug has a higher incidence of severe dermatologic toxicity, such as nivolumab + ipilimumab + platinum (79.1%), nivolumab + ipilimumab (72.9%), camrelizumab + platinum (64.9%), atezolizumab + platinum (47.4%). Pembrolizumab single drug induced dermatologic toxicity incidence (75.2%), followed by nivolumab (44.2%), durvalumab (40.5%), et al 29. Similarly, another network meta-analysis, include 12 RCTs including 8,453 NSCLC patients found that the incidences of severe dermatologic irAEs was ranked nivolumab + ipilimumab (97.4%), pembrolizumab (80.1%), nivolumab (67.1%), pembrolizumab + platinum (43.3%) 30. Another meta-analysis of irAEs with pembrolizumab and nivolumab for the treatment of multiple solid tumors reported an incidence of 16.7% and 14.3% for all grades of rash, respectively 27. A case report by Patil and colleagues described a patient with metastatic urothelial carcinoma who experienced a maculopapular rash during treatment with a PD-L1 inhibitor 31. Interestingly, a meta-analysis that included 137 randomized controlled studies identified the development of vitiligo to be related to improvements in progression-free survival and overall survival time 32. According to a retrospective study, the presence of spongiotic dermatitis and lichenoid eruptions in patients receiving PD-1/PD-L1 inhibitors may indicate a strong immune response and improved oncological outcomes 33. Although most of these dermatologic-related irAEs are self-limiting, early identification and management are essential to prevent progression, improve the quality of patient survival, and prevent interruption of treatment. A number of indicators are available for earlier identification of such dermatologic-related irAEs. The principal constituent of tumor-infiltrating lymphocytes is CD163^+^ macrophages, which have the capacity to generate diverse chemokines by activating cancer-specific stromal factors, including IL-4, POSTN, and RANKL 34. The dermis of various inflammatory cutaneous disorders, including autoimmune conditions like pemphigus vulgaris, scleroderma, and herpetiform aspergillosis, contains POSTN, which is an extracellular matrix protein 35. Increased tumor mesenchymal POSTN expression in melanoma, and POSTN stimulates CXCL5 release from tumor-associated macrophages 36. In a study conducted by Taku and colleagues, 46 patients with advanced melanoma who were treated with nivolumab were examined for their serum sCD163 and CXCL5 levels. The researchers observed that the patients who developed irAEs had significantly elevated absolute serum sCD163 values 37. Despite the lack of significant changes in serum CXCL5 levels, the absolute value of CXCL5 can be utilized as a supplementary marker for the absolute value of serum sCD163. Therefore, both serum sCD163 and CXCL5 levels can be valuable predictors of dermatologic-related irAEs in the context of nivolumab therapy. In addition, bullous pemphigoid (BP), another common type of dermatologic irAEs, appears to be the most commonly expressed antigen in NSCLC 38. According to a report by Hasan and colleagues, NSCLC patients with higher levels of anti-BP180 IgG exhibited improved treatment response and overall survival, as well as an increased risk of cutaneous irAEs when undergoing anti-PD1/PD-L1 therapy 39. Another study on pembrolizumab-induced BP showed that 20 of the 22 cases tested, the serum anti-BP180 autoantibodies were positive. However, in 10 cases (91.90%, 10/11) the circulating autoantibodies of anti-BP230 were negative 40. IgE anti-BP180 were detected in 21 out of 36 BP serum samples, while IgE anti-BP230 were identified in 18 out of the 36 BP sera. Interestingly, IgG and IgE anti-BP180 antibodies were found to be associated with disease activity, whereas IgG and IgE anti-BP230 autoantibodies did not exhibit such a correlation 41. Furthermore, the serum anti-BP180 IgG exhibited higher levels of sensitivity and specificity compared to anti-BP230 IgG 42. It follows that anti-BP180 is more highly expressed in BP and correlates with disease activity. Therefore, the detection of BP180 IgG autoantibody levels is a guide to predict dermatologic irAEs. In addition, a case of psoriasiform dermatologic toxicity caused by pembrolizumab reported by Johnson et al. regressed after systemic interleukin IL17A blockade treatment, suggesting that IL17A has the potential to be one of the predictive markers of dermatologic -related irAEs 43.

### Gastrointestinal adverse events

Immune checkpoint inhibitor-associated colitis has been the most extensively documented. ICIs combination with chemotherapy drug has a higher incidence of colitis, such as atezolizumab + platinum (77.1%), pembrolizumab + platinum (41.4%), however, ICIs alone, nivolumab resulted in the highest incidence of colitis (67.3%), followed by pembrolizumab (60.5%) and durvalumab (45.2%) 30. Another meta-analysis yielded similar results, multi-drug combination: nivolumab + ipilimumab + platinum (72.4%), atezolizumab + platinum (56.9%), pembrolizumab + platinum (38.6%), ICIs alone: nivolumab (63.1%), durvalumab (56.6%), pembrolizumab (54.9%) 29. The most prevalent clinical manifestation of ICI-associated colitis is diarrhea, accompanied by additional symptoms such as fever, abdominal pain, vomiting, blood in the stool, nausea, loss of appetite, and others 44. A combined analysis of CheckMate 817, CheckMate 568, and CheckMate 227, evaluated the safety of first-line treatment with Nivolumab and Ipilimumab in metastatic NSCLC patients. The results indicated that in the patient population treated with the combination of nivolumab and ipilimumab, 78% experienced diarrhea as the most prevalent treatment-related adverse event (TRAE) of all grades (20%), followed by fatigue (18%) and pruritus (17%). TRAE, a treatment-associated adverse event, commonly known as an adverse event (AE) or adverse effect, is an undesired and detrimental consequence or secondary effect that arises due to a medical treatment, procedure, medication, or intervention. These occurrences can span the spectrum from mild and short-lived discomfort to severe and potentially life-threatening reactions. Adverse events have the potential to manifest in a variety of medical contexts, such as clinical trials, medical treatments, and the use of medications. The most frequent grade 3 or 4 TRAEs are increased lipase (6%), fatigue (2%), and diarrhea (2%) 45. Typically, PD-1/PD-L1 inhibitors have a lower occurrence of severe gastrointestinal adverse effects than anti-CTLA-4 drugs 21, 46. Based on a study involving 198 patients with renal cell carcinoma (RCC) or metastatic melanoma (MM) treated with CTLA4 inhibitors, small bowel colitis was identified as the primary major toxicity, observed in 21% of patients 47. Watery diarrhea is commonly related to anti-CTLA-4 therapy (27-54%) 48. A different study conducted in Japan involved 661 cancer patients, which revealed the occurrence of gastrointestinal irAEs (GI-irAEs). Among patients treated with anti-PD-1/PD-L1 antibodies, the incidence of GI-irAEs was 5.6%. In contrast, patients who received anti-CTLA-4 antibodies alone or in combination with anti-PD-1 and anti-CTLA-4 antibodies experienced a higher frequency of GI-irAEs, with a recorded incidence of 16.1%. Interestingly, MM patients with GI-irAEs had better OS with continued ICI treatment 49. Severe immunodigestive adverse reactions are a common cause of ICI discontinuation. In addition, some rare but severe death-causing intestinal perforations have been associated with ICI-induced colitis or small bowel colitis 48. Therefore, early diagnosis is essential to prevent the persistence or progression of colitis and to minimize the interruption of ICI treatment, and exploring predictive markers of ICI-associated GI-irAEs is a key.

The regulation of flora-gut barrier homeostasis by immunotherapy occurs through the apoptosis of intestinal epithelial cells (IECs) mediated by intraepithelial lymphocytes (IELs) 50. Dysbiosis of the intestinal flora is the cause of GI-irAEs, in which the products of the microbiome trigger innate immune responses and activate auto-reactive immune cells 51. Several intestinal bacteria have been shown to enhance the induction of Treg cells and thus maintain intestinal tolerance 52. Increasing evidence suggests a potential correlation between gut microbiome traits and the incidence of ICI-associated colitis as well as tumor response to ICI treatment 44. However, Chaput et al. performed a prospective study which demonstrated that ipilimumab has no impact on the microbiome's composition 53. Fusarium, a specialized anaerobic bacterium, has a role in maintaining the integrity of the colonic mucosa, and its enrichment might be related to the development of colitis 53, 54. Furthermore, it has the potential to elevate CTL concentrations within the tumor microenvironment, leading to prolonged overall survival and increased progression-free time 54. The increase in Bacteroides fragilis, Phascolarctobacterium genus, Enterobacteriaceae family, and Akkermansia muciniphila are thought to be a protective factor for combined immunotherapy for cancer (CIC) and it exerts anti-inflammatory effects in the gastrointestinal tract 55-57. On the contrary, Firmicutes phylum and Veillonela are a risk factor for ICIs-related colitis 53, 58, 59. Bacteroides fragilis produces polysaccharide A to activate the CTLA-4 pathway, which plays a crucial role in immune regulation by upregulating IL-10 levels and ultimately reducing inflammation 60. Furthermore, studies have indicated that blocking CTLA-4 can promote the proliferation of S. fragilis in the colon 61. Additionally, an elevation in the population of Bacteroides fragilis may lead to a further decrease in tumor size among patients undergoing ipilimumab treatment 62. Therapeutic effects of anti-CTLA-4 inhibitors can be restored via relay transfer of Bifidobacterium fragilis-specific T cells into germ-free mice 63. Clinical trials using PD-L1 inhibitors have revealed that Bifidobacterium is capable of facilitating antitumor effectiveness, with oral administration of Bifidobacterium alone improving melanoma tumor control to a similar extent as PD-L1-specific antibody therapy, with combination therapy virtually eliminating tumor growth 64. Interestingly, Bifidobacterium was able to attenuate Treg cell-mediated intestinal inflammation without impairing the antitumor response 65. Wang et al. developed a model of colitis associated with ICIs and observed a reduction in the levels of Lactobacillus in the fecal samples of ICI-treated mice compared to the control group 66. Similarly, a mouse model constructed using trinitrobenzenesulfonic acid showed that Lactobacillus royale strains suppressed intestinal inflammation 80. In addition, Lactobacillus royale enhanced dendritic cell function and improved antitumor response after blockade of PD-L1^67^. Thus, the development of ICI-associated colitis may be influenced by the intestinal flora's type and relative abundance. Certain specific microorganisms may coordinate the initiation of inflammation, while other subgroups may perpetuate ICI-associated colitis. Specific bacteria may be biomarkers of ICI-associated colitis, and further exploration of the complex symbiotic relationship between ICI-induced colitis and bacteria is needed with the ultimate goal of manipulating the microbiota for tumor response and irAEs balance.

Furthermore, a combination of multiple molecular markers, including heightened levels of IL-17, neutrophils, eosinophils, and leukocytes, has been utilized to forecast the occurrence of ICI-induced colitis 68, 69. Patients with ICI-associated colitis were observed to have lower baseline levels of granulocyte colony-stimulating factor (GCSF) 69, 70. Shahabi et al 71 studied the whole blood gene expression profile of 162 patients treated with ipilimumab. Increased therapeutic expression of CD177 and CEACAM1, which are two indicators of neutrophil activation, was found to be strongly associated with GI-irAEs, suggesting that neutrophils may play a role in ipilimumab-associated GI-irAEs. Given the limited sensitivity of these biomarkers, they cannot be utilized in isolation for predicting the occurrence of GI-irAEs in patients. Therefore, it is essential to investigate these biomarkers further in a more extensive patient group.

### ICI-associated hepatitis

The incidence of immunotherapy-induced hepatitis is relatively low, ranging from 5-10% in monotherapy ICI cases. Severe toxicity associated with this form of treatment is less than 2%. However, when combination therapy involving ipilimumab and nivolumab is used, the incidence of immunotherapy-related hepatitis increases substantially, affecting approximately 30% of patients 72, 73. According to research data, the likelihood of experiencing hepatotoxicity is higher in patients treated with ipilimumab (ranging from 3% to 9%) compared to those treated with anti-PD-1/anti-PD-L1 antibodies (ranging from 1% to 2%) 74. In a meta-analysis of 17 RCTs, it was found that CTLA-4 inhibitors (such as ipilimumab and tremelimumab) had a higher risk of total and advanced hepatotoxicity when compared to control regimens. On the other hand, PD-1 inhibitors (like nivolumab and pembrolizumab) were related to a lower risk of total and advanced hepatotoxicity compared to control regimens 75. Besides, the incidence of immune hepatitis varies with use of different drugs and for different cancer types. Alanine aminotransferase (ALT) and Aspartate aminotransferase (AST)are usually elevated in ICI-associated hepatitis 76. The incidence of hepatotoxicity due to ICIs has been reported to be 1% to 17% in melanoma 77. A comparative safety trial of nivolumab in combination with ipilimumab versus ipilimumab in untreated melanoma found that any-grade abnormal elevations in AST and ALT is 22% and 21%, respectively, in the combination nivolumab and ipilimumab group, with greater than grade 3-4 accounting for 11% and 7%. In contrast, the ipilimumab group had only 4% of any-grade AST and ALT elevations and no grade 3-4 toxicity 78. In contrast, a recent study from Japan enrolled a total of 202 patients with multiple cancers who received monotherapy with different ICIs (nivolumab, pembrolizumab, ipilimumab, atezolizumab, and avelumab). The resultant incidence of immune hepatitis was 8.4% (17/202), of which the incidence of grade ≥3 immune hepatitis was 4% (8/202) 79. Incorporating data from 11 clinical trials and 7,086 patients diagnosed with NSCLC, a meta-analysis revealed that the use of ICIs resulted in an overall incidence rate of 6.18% for ALT elevation, 4.99% for AST elevation, and 1.09% for hepatitis 80. Several reports have shown that the median time to onset of immune hepatitis is approximately 6-14 weeks after use of ICIs and the median time of 10 weeks from the severe immune-related hepatitis 81. However, the exact time is associated with the type of tumor and the type of drug used. For example, lung cancer patients treated with nivolumab developed hepatitis later than those treated with pembrolizumab, and also later than those treated with melanoma 82, 83. Since the time of appearance of immune hepatitis is not quite the same as given in different reports, it is not reliable to identify whether it is immune hepatitis only by the time of appearance.

Although the precise mechanism behind ICI-induced hepatitis is not completely comprehended, it is believed to entail a multifaceted interaction between the immune system and liver cells. Here are some possible mechanisms: (1) activation of T cells: ICIs activate T cells, which are a type of immune cell that can attack cancer cells. However, in some cases, these activated T cells may also attack healthy liver cells, leading to hepatitis 84-86. (2) Dysregulation of cytokines: ICIs can cause a dysregulation of cytokines, which are proteins that regulate the immune response. This dysregulation can lead to an overactive immune response against liver cells 87, 88. (3) Direct toxicity: Some ICIs may have direct toxic effects on liver cells, leading to hepatitis 89-91. (4) Pre-existing liver damage: Patients with pre-existing liver damage, such as hepatitis B or C, cirrhosis, or fatty liver disease, may be more susceptible to immunotherapy-related hepatitis 92, 93. (5) Genetic factors: Certain genetic factors may increase the risk of developing immunotherapy-related hepatitis, although the exact genes involved are not yet known. For example, genetic variants such as HLA-DRB107, HLA-DQA102, HLA- DRB1*04:01 and HLA- DRB1*15:01-DQB1*06:02 have been associated with the development of immunotherapy-associated hepatitis 94-96. It is essential to note that the exact mechanism of immunotherapy-related hepatitis may vary depending on the specific type of immune checkpoint inhibitor used, as well as individual patient factors.

There are fewer reports on predictors of immune hepatitis. Some studies have shown that female patients treated with ICIs have a higher probability of developing grade ≥3 immune hepatitis compared to men 79. Certain underlying conditions might enhance the risk of immunotherapy-associated hepatitis. For example, patients with autoimmune diseases or infectious diseases may be more likely to develop immunotherapy-associated hepatitis 97. ICI-associated hepatitis shares some similarities with classical autoimmune liver disease (AILD). In their study, Coukos et al. identified three distinct histological patterns of liver injury caused by ICIs treatment: hepatitic (52%), cholangitic (19%), and mixed (29%). Furthermore, when comparing the ICIs-associated group to the AILD group, the former showed a higher prevalence of centrilobular injury and granuloma formation. Besides, CD8^+^ T cells tended to be increased in ICIs-related hepatitis 98. Patients with poor liver function are more likely to develop immunotherapy-associated hepatitis. In addition, diet and lifestyle may also influence the development of immunotherapy-associated hepatitis. For example, excessive alcohol consumption and obesity may increase a patient's risk of developing immunotherapy-associated hepatitis 99, 100. In addition, induction of liver injury by ICIs may be related to certain antibodies. Taking NSCLC as an example, research has shown that antituberculous antibody (ANA) and antimitochondrial antibody (AMA) may help predict ICI-induced liver injury. The present study examined NSCLC patients who were administered either nivolumab (3 mg/kg every 2 weeks) (186 patients) or pembrolizumab (200 mg every 3 weeks) (66 patients) as monotherapy. The results indicated that for patients treated with nivolumab, positive ANA testing was identified as a risk factor for ICI-related hepatitis (risk ratio 2.133: 95% CI 1.085-4.194: p=0.0281). On the other hand, for patients receiving pembrolizumab, both positive ANA and age were found to be potential risk factors for ICIS-related hepatitis (risk ratios 7.834: 95% CI 1.743-35.21: p=0.0073 and 0.896: 95% CI 0.896: p=0.0050, respectively) 101. Predicting immune-mediated hepatitis is relatively difficult, and primary liver diseases and other causes of liver function abnormalities should be excluded first. With the gradual application of ICIs in clinical practice, the incidence of immune-related hepatitis is increasing. Accurate prediction of immune-related adverse reactions is particularly important for the prognosis of patients treated with ICIs.

### Endocrine system diseases

ICIs treatment can lead to the development of various endocrinopathies such as hypophysitis, hyperthyroidism or hypothyroidism, thyroiditis, primary adrenal insufficiency (PAI), and insulin-dependent diabetes mellitus (DM) 102. Certain endocrinopathies have been observed to occur more frequently with specific ICIs. For instance, hypophysitis is more prevalent following treatment with anti-CTLA-4 drugs like ipilimumab, while thyroid dysfunction is more commonly associated with anti-PD-1 drugs such as nivolumab and pembrolizumab. Moreover, the co-administration of these drugs might increase the risk of developing ICI-associated endocrinopathies 102. In a meta-analysis comprising 34 studies examining ICIs therapy, it was found that out of 6472 patients, 85 cases of hypophysitis were observed, with 34 cases classified as grade 3 or higher (constituting 0.5% of the total cases). During treatment, the ipilimumab-nivolumab combination therapy had the highest recorded incidence of pelvic inflammation (6.4%), whereas anti-CTLA-4 therapy had rates of 3.2%, anti-PD-1 therapy had rates of 0.4%, and anti-PD-L1 therapy had rates of less than 0.1% 102. The frequency of hypophysitis caused by anti-CTLA-4 antibodies was found to be between 8.0% to 11.7% 103. Individuals who underwent anti-PD-1 therapy had a significantly lower likelihood of developing hypophysitis in comparison to those who received ipilimumab treatment 104. Thyroid dysfunction represents a commonly observed endocrine-related irAEs associated with the use of ICIs 105. The ICIs-associated thyroid dysfunction is mostly characterized by hyperthyroidism, hypothyroidism, and/or thyroiditis 106-108. Compared to anti-PD-L1 monotherapy or anti-CTLA-4 monotherapy, the incidence of thyroid dysfunction appears to be higher with anti-PD-1 therapy and combined ipilimumab-nivolumab therapy 102. A meta-analysis of anti-CTLA-4, anti-PD-1/PD-L1 treatments that included 37 randomized controlled studies found that 194 of 7531 patients developed hyperthyroidism and 472 of 7551 patients developed hypothyroidism 102. Morganstein et al. conducted the biggest single-center study on thyroid dysfunction associated with ICIs. Their study confirmed the relatively high frequency of thyroid dysfunction in ICI-treated patients, and revealed that this condition was most common in patients who received the ipilimumab-nivolumab combination therapy 109. According to case series reports, the detection of thyroid dysfunction associated with ICI occurred within a median range of 18 to 123 days from the start of ICIs treatment 110-112. PAI is a rare irAEs relate to ICIs treatment and only a few cases of ICI-related PAI have been reported 113-116. Although rare, ICIs-associated DM can be a life-threatening irAEs. The overwhelming majority of reported cases of diabetes mellitus associated with ICIs are linked to anti-PD-1 therapy. However, there have been a few reported instances of diabetes associated with anti-PD-L1 therapy as well 117-119. Besides, anti-CTLA-4 monotherapy seems to have an exceptionally low incidence of leading to ICIs-associated DM 120.

The exact pathogenesis of ICIs-associated endocrinopathies is unclear. The incidence of ICI-associated endocrinopathies increases with dose and varies according to ICI therapy. Earlier prediction can improve patient survival and it is especially important to identify predictive markers. According to Nakamura et al., there is a positive correlation between absolute eosinophil counts >240/µL and relative eosinophil counts >3.2%, and the onset of endocrine irAEs 121. Both in vitro experiments and studies using mouse models have demonstrated that antibody-dependent cell-mediated cytotoxicity (ADCC) and complement pathways play a role in the development of hypophysitis induced by ipilimumab 122, 123. According to Tahir et al., alterations in the ploidy of autoantibodies against GNAL or ITM2B following ICIs treatment were linked to the subsequent occurrence of hypophysitis. The changes in anti-GNAL and anti-ITM2B autoantibodies were distinct from the values observed in patients who did not develop hypophysitis 104. Previous studies have comprehensively analyzed indirect immunofluorescence (IIF) results and found significant differences in the distribution of cytoplasmic staining patterns between pituitary disorders, with granular cytoplasmic staining being highly predictive of pituitary autoimmunity. Moreover, in patients with positive IIF assays, purified IgG produced clearer signals, which improved the sensitivity and specificity of the assay 124. High rate of antipituitary antibody (APA) positivity in hypophysitis induced by anti-CTLA-4 antibodies and the presence of anti-PD-1 therapy in hypophysitis is not yet known 125. Antihypothalamic antibody (AHA) is frequently detected in patients receiving immunotherapy, suggesting its possible is a biomarker for early detection of pituitary disease associated with ICIs. In a cross-sectional study involving 54 cancer patients undergoing anti-PD-1 or anti-PD-L1 therapy and 50 healthy controls, there was a statistically significant rise in APA observed among these patients 126. Furthermore, the impact of the host's germline on the response to ICI therapy is worth exploring, and human leukocyte antigen class I (HLA-I) serves as a valuable prognostic biomarker for this purpose 127. CD8^+^ T cells play a major role in the antitumor activity of ICIs and are required to present tumor antigens via HLA-I 128. One study evaluated HLA-Cw12, HLA-DR15, HLA-DQ7 confirmed that HLA-DQ7 is related to the development of ICI-associated isolated adrenocorticotropic hormone deficiency (IAD) in the absence of pituitary enlargement 129. HLA-DR4 was present in 76% patients of insulin-dependent diabetes induced with ICIs 130, 131. Besides, the class II trait is HLA HLA-DR4-DR53 and HLA-DR15 in ICI-induced thyroiditis patient 132.

Furthermore, elevated thyroperoxidase (TPO) antibodies and thyrotropin receptor antibody (TgAb) was found in many cases of thyroid dysfunction associated with ICIs 107, 112, 133, 134. However, the role of thyroid autoantibodies in the pathogenesis of ICIs-associated thyroid dysfunction, as well as whether elevated levels raise the risk of such dysfunction, remains uncertain. Osorio et al. had reported that elevated thyroid autoantibody levels after ICIs treatment did not guarantee significant thyroid dysfunction 108. However, Maekura et al. found that baseline levels of TPO Ab and TgAb may predict hypothyroidism due to nivolumab treatment of NSCLC. Out of the 64 patients who participated in this trial, 5 (7.8%) developed hypothyroidism following treatment with nivolumab. Patients who developed primary hypothyroidism were found to have significantly positive TPO antibody and TgAb levels 135. Further studies are required to confirm whether increased levels of TPO antibodies and TgAbs at baseline are risk factors for ICIs-related thyroid dysfunction. A higher risk of developing atezolizumab-induced thyroid dysfunction, which is linked to improved survival rates, has been observed in individuals with elevated polygenic risk scores (PRS) for thyroid autoimmunity-associated genetic variants 136. An analysis in the field of bioinformatics discovered that, in cases of hypothyroidism resulting from CTLA-4 treatment, genes such as ALB, MAPK1, SPP1, PPARG, and MIF are found to be overexpressed. On the other hand, in cases of hyperthyroidism caused by the same treatment, genes such as ALB, FCGR2B, CD44, LCN2, and CD74 are overexpressed. These genes may potentially serve as biomarkers to predict immunotherapy-induced thyroid dysfunction 137. Although the exact mechanism of ICI-associated endocrinopathies remains unclear, these findings indicated that autoantibodies may be potential biomarkers for prediction and treatment of ICI induced endocrinopathies and deserve further investigation.

### ICIs-associated Pneumonitis

The most prevalent manifestation of pulmonary toxicity in patients receiving ICIs is pneumonia 138. The Society for Immunotherapy of Cancer's (SITC) Toxicity Management Working Group reports that targeted therapy of PD-1/PDL-1 and CTLA-4 is associated with pneumonitis in less than 5% of cases overall, with severe complications (grade three or higher) occurring in only 1-2% of patients 138. In a meta-analysis of 20 RCTs investigating PD-1 inhibitors across different tumor types (including 12 melanoma studies, five NSCLC studies, and three RCC studies), which involved a total of 4496 unique patients, the occurrence of grade three or higher pneumonia was found to be between 0% to 4.3% and 0% to 10.6% for any grade of pneumonia. Four studies reported 1 to 3 pneumonia-related deaths (0.2% - 2.3%). Compared to any grade of pneumonia in melanoma, NSCLC had a significantly higher incidence of pneumonia (4.1% vs. 1.6%; P=0.002), as well as a higher incidence of grade ≥3 pneumonia (1.8% vs. 0.2%; P<0.001). Furthermore, the combination therapy group had a significantly higher incidence of morbidity (6.6% vs. 1.6%; P<0.001) and grade ≥3 pneumonia (1.5% vs. 0.2%; p=0.001) compared to the monotherapy group 139, 140. Meta-analysis of published anti-PD-1/PD-L1 and anti-CTLA-4 trials assessing their incidence using over 16 million adverse drug reaction data from the WHO pharmacovigilance database and records from 7 academic institutions showed widespread central pneumonia with anti-PD-1/PD-L1 monotherapy (115 [35%]), compared with pneumonitis with ipilimumab monotherapy (15 [8%]) 141. The risk of pneumonia is higher with anti-CTLA-4/anti-PD-1/PD-L1 combination therapy compared to ICI monotherapy 140, 142. Extensive studies are required to identify the risk factors related to ICI-associated pneumonia (IAP), which should include investigating the association between smoking history and the risk of developing pneumonia, as well as exploring the role of the PD-1/PD-L1 pathway in the pathogenesis of pneumonia and the identification of predictive markers.

A retrospective study included 430 patients with anti-PD-1 antibodies in combination with or without chemotherapy or anti-angiogenic therapy showed a prevalence of 15.6% for IAP and 3.7% for severe IAP. Patients with IAP had low eosinophil percentages at endpoint (E_end_)/ baseline (E_bas_) values (p = 0.001), especially for severe IAP (p = 0.036). In addition, E_end_/E_bas_ values performed well in the diagnosis of severe IAP (AUC:0.800, p<0.001), indicating that eosinophil percentage correlates with the diagnosis, prediction and prognosis of IAP 143. A meta-analysis of 17 RCTs with 2758 patients revealed that individuals with NSCLC and interstitial lung disease (ILD) had a higher likelihood of developing pneumonia after immunotherapy. Subgroup analysis indicated that ILD, radiation pneumonitis, and interstitial lung abnormalities were predictors of IAP, along with the neutrophil lymphocyte ratio (NLR) and the actual eosinophil acidic granulocyte count, which may have potential predictive value for IAP 144. Continuous monitoring of NLR trends may be indicative of the onset and severity of pneumonitis as well as the prognosis, according to these findings 145. The presence of homozygosity at one or more HLA-I loci was associated with a decreased likelihood of developing pneumonia or experiencing toxicity of grade 3 or higher 146. Both baseline and dynamic levels of IL-10 in the plasma are significantly and autonomously related to an increased risk of pneumonia, and may serve as useful clinical markers to monitor pneumonitis in patients undergoing ICIs therapy 147.

### Cardiovascular toxicity

The most common cardiovascular irAEs in ICIs therapy is myocarditis, which initially surfaced in case reports, as well as in phase II and III clinical trials of ICIs, and subsequently in case series 148-152. The incidence rate of myocarditis presented range from 0.5 to 1.7% 10. In comparison to other irAEs, myocarditis exhibits the highest fatality rate at 39.7% 153, 154. In a study of 964 patients treated with ICIs, Mahmood and colleagues discovered that the incidence of myocarditis was 1.14%, with a median onset of 34 days 155. Pharmacovigilance reports indicate that myocarditis was detected in 0.06% of patients treated with single nivolumab therapy, whereas in patients treated with a combination of ipilimumab-nivolumab therapy, the incidence was 0.27% 151. However, myocarditis is more frequent than initially reported in early ICIs clinical trials 156. According to a recent review of the WHO database, the higher incidence of ICIs-related myocarditis reported in recent multicenter registries may more accurately reflect the true incidence 157. In a recent randomized trial comparing nivolumab to combined relatlimab and nivolumab, where an incidence rate of myocarditis is 0.6% for single nivolumab and 1.7% for combination relatlimab and nivolumab 10. In addition to myocarditis, other cardiovascular irAEs are increasingly being reported, such as vasculitis pericardial disease, arrhythmia, acute coronary syndrome, thrombotic events, and left ventricular (LV) dysfunction, without evidence of myocarditis 158-161.

Although the incidence of ICIs-associated myocarditis is relatively low, its lethality is high; therefore, early diagnosis of myocarditis, even in the subclinical phase without clear clinical signs and symptoms, enables timely intervention 162. And biomarkers as the best diagnostic tool can predict early (e.g., administered at baseline) or monitor early (e.g., administered continuously during chemotherapy) the onset of myocarditis. Nearly all patients with ICIs-related myocarditis have been shown to exhibit elevated hypersensitivity troponin levels, as per several studies 163. Persisting inflammation of the myocardium is suggested by elevated high troponin levels 164. A strong association between troponin levels and the risk of major adverse cardiac events, including cardiovascular death and shock, has been observed in patients with ICIs-associated myocarditis.

Johnson and colleagues' report indicates that the myocarditis linked with ICIs involves the infiltration of CD4+ and CD8+ T cells, along with CD68+ macrophages, into the myocardium and conduction system. Notably, the presence of other immune cells, such as B cells, was not observed 151. TNF-α, a cytokine with pro-inflammatory properties, has been shown to induce acute and chronic hemodynamic changes and left ventricular dysfunction. Overexpression of TNF-α in cardiomyocytes can lead to cardiomyopathy and myocarditis 165. Pro-inflammatory cytokines, such as IL-1B, IL-2, and IL-6, have negative inotropic and cytotoxic effects on cardiomyocytes 166-168. The lack of histological analysis makes it uncertain whether classical myocarditis, characterized by myocardial immune infiltration and subsequent myocardial cell death, occurs in cytokine release syndrome. As cancer immunotherapy advances, it becomes more crucial to comprehend the impact of pro-inflammatory cytokines on the cardiovascular system 151, 153, 169. However, the few clinical trials in which baseline troponin was measured to predict myocarditis after ICIs initiation did not demonstrate good predictive power or specificity 170. In one of the largest prospective studies conducted to date via Waliany et al. 11.2% of the 214 patients included were found to have positive high-sensitivity troponin i values (≥55 ng/L) compared to a 1.4% incidence of myocarditis (3 cases) in 2021. Thus, in most cases, troponin I positivity was attributed to cardiovascular causes other than myocarditis 171. Zhang et al. found that cardiac biomarkers, including mean cardiac troponin T and NT-proBNP, were significantly elevated after treatment with ICI, peaked at about one month, and then gradually decreased 172. Therefore, specific troponin thresholds for ICI-associated myocarditis need to be validated by larger clinical trials, and the effectiveness of troponin-based screening methods in obtaining timely diagnosis and risk of inappropriate treatment interruption still needs to be proven 170, 173. Similarly, in cases of ICIs-related myocarditis, serum creatine kinase (CK), creatine kinase isoenzyme (CK-MB) and N-terminal pro-B type natriuretic peptide (NT-proBNP) were elevated, but specificity was as low as that of troponin 174, 175. In their study, Boughdad and colleagues enrolled patients who were clinically suspected of having associated myocarditis and discovered that the majority of those who were tested (six out of seven) showed elevated levels of serum inflammatory cytokines such as IL-6, as well as chemokines CXCL9, CXCL10, and CXCL13. In the study, it was observed that four out of five patients with myocarditis displayed an imbalance between Th1 and Th2 immune cells, which resulted in significant inflammatory responses from Th1, Th1/Th17, and Th17 CD4 memory T-cells. Additionally, four to five patients had a notable increase in non-classical monocytes and reduced levels of CD31. Further investigation is necessary to determine if the aforementioned inflammatory cytokines can serve as indicators for immunotherapy-associated myocarditis 176. Zhu et al. used time-of-flight mass spectrometry flow cytometry to investigate peripheral blood mononuclear cells of 52 patients who underwent immunotherapy. Their findings demonstrated a significant rise in clonally cytotoxic Temra CD8+ cells in the bloodstream of eight patients with ICI myocarditis, accompanied by distinct transcriptional alterations in these expanded effector CD8^+^ cells. Notably, the chemokines CCL5/CCL4/CCL4L2 were found to be up-regulated, and could be promising targets for diagnosis and treatment to lower the occurrence of life-threatening cardiac iRAEs in ICI-treated cancer patients 177. Zhang et al. found that cardiac biomarkers, including mean cardiac troponin T and NT-proBNP, were significantly elevated after treatment with ICIs, peaked at about 1 month, and then gradually decreased 172. In summary, ICIs may cause various adverse cardiovascular events (ACEs) at a higher rate than in patients receiving only conventional chemotherapy. Regular electrocardiography, echocardiography and cardiac biomarkers can help to detect ACE caused by ICIs early and treat it timely 172.

### Neurological irAEs

Among the 3,763 patients with advanced melanoma who received nivolumab with or without ipilimumab in the global pharmacovigilance and epidemiology database, only a small proportion experienced neurological irAEs (NirAEs). Specifically, 35 patients (0.93%) developed a total of 43 serious neurological irAEs, which included neuropathy (n = 22), noninfectious meningitis (n = 5), encephalitis (n = 6), and others 178. The most common NirAEs is neuromuscular disease (approximately 5.5%) 179, with the most prevalent neuromuscular syndrome being myositis, which occurs in nearly 3% of patients treated with anti-PD-1/PD-L1. In contrast, central nervous system (CNS) involvement is notably rare, with a frequency of only 0.5%, and usually presents as encephalitis, meningitis, vasculitis, myelitis, and cranial neuropathy 180. Other studies have reported several neurological irAEs, including peripheral neuropathy (1.3%), myasthenia gravis (1.2%), myelitis (0.8%), meningitis (0.4%), encephalitis (0.3%), and Guillain-Barre syndrome (<0.1%) 181, 182. Despite the prevalence of evidence, most of the evidence collected is from small case reports or case series. Most events are mild and accompanied by nonspecific symptoms, such as headache; <1% of patients have a grade 3 or higher irAEs 181. The collective occurrence rates are less than 4% for anti-CTLA-4 antibody therapy, approximately 6% for anti-PD-1 antibody therapy, and around 12% for a combination of both anti-CTLA-4/PD-1 antibody therapy 181. In contrast, relapsed/refractory large B-cell lymphoma (R/R LBCL) patients who received CD19-directed chimeric antigen receptor T-cell therapy (CAR-T) experienced a notably higher occurrence of immune effector cell-related neurotoxicity syndrome (ICANS). Of the patients, 25 (56%) developed ICANS, with 18 (72%) experiencing severe ICANS (CTCAE grade 3-4). The median duration of ICANS was 5 days (range, 3-11). However, neither the advancement of ICANS nor its treatment correlated with worse CR, PFS, or OS 183.

Although the incidence of NirAEs is low, the potential severity and consequent interruption of cancer treatment that they can entail makes early detection particularly essential 184. Studies have shown that elevated levels of IL17A are correlated with NirAEs^185^. Elevated CK levels are commonly observed in patients diagnosed with neuromyositis optica, particularly in those who receive a combination of ICIs. Although some studies suggest that CK levels are higher in individuals with severe disease as opposed to mild disease, there is no clear association between CK levels and symptom severity 186-188. Oligoclonal bands, polycythemia and hyperproteinemia in cerebrospinal fluid are nonspecific inflammatory alterations that are usually found in patients with immune-mediated encephalitis 189. There have been reports indicating that heightened levels of adenosine deaminase (ADA) can serve as a valuable diagnostic marker for ICIs-associated CNS toxicity 190. In summary, these predictive markers may serve as a predictive and therapeutic target, but relatively poor specificity and further research is necessary to substantiate this hypothesis.

### Rheumatic irAEs

Although the literature on rheumatic irAEs (Rh-irAEs) in patients treated with ICIs is growing rapidly 191, 192, the incidence has not been well appreciated, especially in some cases where Rh-irAEs resembles various rheumatic diseases, Such as arthritis, Sicca and Sjogren's s (SS), systemic lupus erythematosus (SLE), myositis, vasculitis, and polymyalgia rheumatic (PMR) 193-197. The European League Against Rheumatism (EULAR) led by screening 630 references and including 22 retrospective and prospective studies found that the prevalence of Rh-irAEs ranging from 1.5% to 22% in the real world, suggests that Rh-irAEs is underreported in clinical trials 198-202. A systematic review consisting of 33 clinical trials, three observational studies, and 16 case reports or series on rheumatic and musculoskeletal irAEs found that the occurrence rates of arthralgia ranged from 1% to 43%, while the prevalence rates for myalgia were between 2% to 20%. These results underscore notable variations in symptom reporting and diagnosis of rheumatic irAEs 191. The reasons for this discrepancy may be multifaceted. Firstly cancer-related adverse events in clinical trials are reported using multiple mutually exclusive musculoskeletal symptom codes. For example, arthritis may be coded as arthritis, arthralgia, joint swelling, or pain in the extremities 203. Second, many clinical trials do not report Rh-irAEs or partially report only highly and/or frequently occurring adverse events (i.e., in 10% of patients). After ICI treatment, patients may develop arthritis or arthralgia, with incidence rates of 3-9% for anti-CTLA-4, 7-11% for anti-PD-1/PD-L1, and 11% for combination therapy 204, 205. According to a prospective observational study conducted at a single center involving 524 patients treated with ICI, 35 (6.6%) of the participants developed Rh-irAEs. The Rh-irAEs included inflammatory arthritis (3.8%), non-inflammatory musculoskeletal disorders (2.8%), as well as specific conditions like rheumatoid arthritis, PMR, or psoriatic arthritis 206. The most significant Canadian multicenter cohort study to date, consisting of 117 patients with Rh-irAEs, revealed that the most prevalent Rh-irAEs was symmetrical polyarthritis. The cohort also reported other Rh-irAEs, including non-inflammatory musculoskeletal symptoms, PMR, and myositis 207. A retrospective study conducted at a single center and involving 264 patients with different types of cancer who received ICI, revealed that 16.3% (n=43) of these patients developed at least one Rh-irAEs. Interestingly, patients who developed Rh-irAEs had a significantly higher median overall survival of 132 weeks as compared to those who did not develop Rh-irAEs, whose median overall survival was 42.7 weeks, with a P value of less than 0.01. This significant difference was observed even after adjusting for various factors in a multivariate analysis, P < 0.05), indicating that Rh-irAE development in ICI therapy is linked with a better prognosis 208. Therefore, it is crucial to detect Rh-irAEs early to enable prompt and optimal management, considering the long-term response potential of these patients.

Current information on the incidence of Rh-irAEs is found in reports of observational studies. There are few studies investigating possible underlying immunopathogenic mechanisms. Until now, the majority of reports on Rh-irAEs have not identified typical autoantibodies, traditional HLA correlations, or any other biomarkers of unexplained illness. However, there are some markers that have demonstrated the ability to predict Rh-irAEs. Disease activity in antinuclear cytoplasmic antibody (ANCA)-related vasculitis and SLE could be linked to T-cell exhaustion, as an instance 209. In addition, in rheumatoid arthritis patients who test positive for anti-cyclic citrullinated peptide (ACPA), ICI treatment predisposes them to acute exacerbations, especially those receiving anti-PD-1 therapy 210. Elevated levels of inflammatory cytokines such as IL17 and TNF-α have been observed in rheumatic irAEs, and inhibitors targeting these cytokines can be utilized for managing these adverse effects. However, whether these inflammatory cytokines can be used as predictive markers in this regard is still controversial 211, 212, further research in this area is urgently needed.

## Conclusion

The treatment landscape for diverse solid tumors and hematological malignancies has been transformed by cancer immunotherapy, such as ICIs and CAR-T, resulting in increased benefits for a larger number of individuals with cancer. However, an increasing number of adverse reactions caused by ICIs have also been reported. As the clinical use of immunotherapy continues to rise, it becomes crucial to recognize and manage its distinct toxicities. The most demanding issue in clinical practice is presently how to maximize the therapeutic benefits of ICIs while minimizing the issues arising from irAEs. Clinicians should fully understand the diversity and severity of adverse reactions related to immunotherapy drugs, improve their ability to diagnose and treat them early, pay attention to medication details, and make these drugs work better to bring more clinical benefits to patients. Although publications on potential predictive irAEs biomarkers are also rapidly increasing, unfortunately, there are currently no clinically validated and effective precise biomarkers except for some markers with potential predictive ability but low specificity. Hence, it is imperative to conduct extensive and comprehensive prospective clinical studies in the future, along with interdisciplinary collaborations, to delve deeper into the pathogenesis and disease patterns of these irAEs. This would aid in developing appropriate grading criteria, optimal treatment measures, and identifying more specific predictive biomarkers. Additionally, enhancing physicians' proficiency in diagnostic and management skills and formulating relevant treatment guidelines would assist clinicians in improving their early recognition, diagnosis, and treatment of ICIs-related adverse reactions.

## Supplementary Material

Supplementary figure.

## Figures and Tables

**Table 1:** Immune checkpoint inhibitors approved by the Food and Drug Administration and the China National Drug Administration

Drug	Target	Indications		
		FDA		NMPA
Pembrolizumab	PD-1	Melanoma		Melanoma
		Advanced non-small-cell lung cancer		Non-small-cell lung cancer
		Squamous-cell carcinoma of the head and neck		Squamous-cell carcinoma of the head and neck
		Non-muscle invasive bladder cancer		Esophageal squamous cell carcinoma
		Advanced renal-cell carcinoma		Hepatocellular carcinoma
		Advanced colorectal cancer with MSI-H or dMMR		Colorectal cancer with MSI-H or dMMR
		Solid tumors with MSI-H or dMMR		
		High-risk early-stage or advanced triple-negative breast cancer		
		Classic Hodgkin's lymphoma		
		Advanced gastric cancer		
		Advanced endometrial cancer		
		Primary mediastinal B-cell lymphoma		
		Advanced hepatocellular carcinoma		
		Advanced esophageal carcinoma		
		Uroepithelial carcinoma		
		Advanced Merkel cell carcinoma		
		Squamous-cell carcinoma of skin		
				
Nivolumab	PD-1	Advanced non-small-cell lung cancer		Non-small-cell lung cancer
		Advanced Melanoma		Squamous-cell carcinoma of the head and neck
		Squamous-cell carcinoma of the head and neck		Gastric cancer/adenocarcinoma of esophagogastric junction
		Advanced renal-cell carcinoma		Advanced gastric cancer
		Advanced hepatocellular carcinoma		Malignant pleural mesothelioma
		Advanced bladder cancer		Esophageal squamous cell carcinoma
		Classic Hodgkin's lymphoma		
		Colorectal cancer with MSI-H or dMMR		
		Malignant pleural mesothelioma		
		Gastric or gastroesophageal junction cancer or esophageal adenocarcinoma		
				
Cemiplimab	PD-1	Cutaneous Squamous Cell Carcinoma,		
		Non-small cell lung cancer		
				
Toripalimab	PD-1			Melanoma,
				Nasopharyngeal carcinoma
				Esophageal cancer
				Uroepithelial carcinoma
				
Sintilimab	PD-1			Hodgkin's lymphoma,lung cancer
				Esophageal Cancer
				Hepatocellular carcinoma
				Gastric cancer/adenocarcinoma of esophagogastric junction
				Hepatocellular carcinoma
				
Camrelizumab	PD-1			Classic Hodgkin's lymphoma
				Esophageal squamous carcinoma
				non-small-cell lung cancer
				Hepatocellular carcinoma
				Nasopharyngeal carcinoma
				
Tislelizumab	PD-1			Hodgkin's lymphoma
				Lung cancer
				Hepatocellular carcinoma
				Uroepithelial carcinoma
				Solid tumors with MSI-H or dMMR
				
Penpulimab	PD-1			Hodgkin's lymphoma
Serplulimab	PD-1			Solid tumors with MSI-H or dMMR
Pucotenlimab	PD-1			Solid tumors with MSI-H or dMMR
Retifanlimab	PD-1	Metastatic or recurrent locally advanced Merkel cell carcinoma		
Atezolizumab	PD-L1	Extensive stage small cell lung cancer		
		Metastatic non-small-cell lung cancer		
		Advanced triple-negative breast cancer		
		Locally advanced or metastatic uroepithelial carcinoma		
		Advanced Melanoma		
				
Durvalumab	PD-L1	Non-small-cell lung cancer		
		Small cell lung cancer		
		Uroepithelial carcinoma.		
		Cholangiocarcinoma		
				
Envafolimab	PD-L1			Solid tumors with MSI-H or dMMR
Zimberelimab	PD-L1			Hodgkin's lymphoma
Sugemalimab	PD-L1			Hepatocellular carcinoma Non-Small-Cell Lung Cancer
				Solid tumors with MSI-H or dMMR
				
Avelumab	PD-L1	Uroepithelial carcinoma		
		Advanced renal-cell carcinoma		
		Merkel cell carcinoma		
				
Ipilimumab	CTLA-4	Melanoma		
				
Tremelimumab	CTLA-4	Hepatocellular carcinoma		
		Non-small-cell lung cancer		
				
Cadonilimab	PD-1/CTLA4			Cervical Cancer
Relatlimab	LAG-3	Melanoma		

Note: FDA, Food and Drug Administration; NMPA, National Medical Products Administration; PD-1, programmed cell death 1; PD-L1, programmed cell death ligand 1; CTLA-4, cytotoxic T-lymphocyte antigen 4; LAG-3, lymphocyte activation gene-3. Data as of: March 2023.

**Table 2:** The various irAEs in patients with cancer treated with ICIs and potential biomarkers for various irAEs.

irAEs	Type	Potential predictive biomarkers
Dermatologic-related Toxicity	Macular Papules, Pruritus, Vitiligo, Lichenoid Dermatitis, Psoriasis, Herpetiform Aspergillosis, Dermatomyositis, Pemphigus, Acne-like Rash, Vascular Disease-like Changes,	sCD163, CXCL5, BP180 IgG autoantibody, IL17A
Gastrointestinal adverse events	Colitis	Combine of IL-17, neutrophils, eosinophils, and leukocytes, GCSF, CD177, CEACAM1, Intestinal microbiota (Fusarium, Bacteroides fragilis, Phascolarctobacterium genus, Enterobacteriaceae family, Akkermansia muciniphila, Firmicutes phylum, Veillonela, S. fragilis, Bifidobacterium, Lactobacillus.
Hepatic Toxicity	ICI-associated hepatitis	ANA, AMA
Endocrine System Diseases	Hypophysitis, Hyperthyroidism Hypothyroidism, Thyroiditis Primary adrenal insufficiency, Insulin-dependent diabetes mellitus	anti-GNAL, anti-ITM2B, APA, AHA, HLA-I, HLA-DQ7, HLA-DR4, TgAb, ALB, FCGR2B, CD44, LCN2, CD74
Respiratory Toxicity	ICI-associated Pneumonitis	NLR, HLA-I, IL-10
Cardiovascular Toxicity	Myocarditis	CK, CK-MB, NT-proBNP, IL-6, CXCL9, CXCL10, CXCL13, CD31, CCL5, CCL4, CCL4L2
Neurological irAEs	Neuromuscular disease, Neuropathy, Noninfectious meningitis, Encephalitis, Vasculitis, Myelitis, Cranial neuropathy, Peripheral neuropathy, Myasthenia gravis, Guillain-Barre syndrome	IL17A
		CK
		ADA





Rheumatic irAEs	Arthritis, Sicca and Sjogren's, Systemic lupus erythematosus, Myositis, Vasculitis, Polymyalgia rheumatic	IL17, TNF-α,

## Abbreviations

ICIs: Immune checkpoint inhibitors
irAEs: immune-related adverse events
NSCLC: non-small cell lung cancer
PD-1: programmed cell death 1
PD-L1: programmed cell death ligand 1
CTLA-4: cytotoxic T lymphocyte-associated protein 4
LAG-3: lymphocyte-activated gene-3
FDA: Food and Drug Administration
NMPA: National Medical Products Administration
TCR: T-cell receptor
MSI-H: MicroSatellite Instability-High
dMMR: Mismatch repair deficiency
RCTs: randomized clinical trials
BP: bullous pemphigoid
TRAE: treatment-related adverse event
RCC: renal cell carcinoma
MM: metastatic melanoma
IECs: intestinal epithelial cells
IELs: intraepithelial lymphocytes
CIC: combined immunotherapy for cancer
GCSF: granulocyte colony-stimulating factor
ALT: Alanine aminotransferase
AST: Aspartate aminotransferase
AILD: autoimmune liver disease
ANA: antituberculous antibody
AMA: antimitochondrial antibody
PAI: primary adrenal insufficiency
DM: diabetes mellitus
ADCC: antibody-dependent cell-mediated cytotoxicity
IIF: indirect immunofluorescence
APA: antipituitary antibody
AHA: Antihypothalamic antibody
HLA-I: human leukocyte antigen class I
IAD: isolated adrenocorticotropic hormone deficiency
TPO: thyroperoxidase
TgAb: thyrotropin receptor antibody
CK: creatine kinase
CK-MB: creatine kinase isoenzyme
NT-proBNP: N-terminal pro-B type natriuretic peptide
CNS: central nervous system
CAR-T: chimeric antigen receptor T-cell
ICANS: immune effector cell-associated neurotoxicity syndrome
ADA: adenosine deaminase
SS: Sicca and Sjogren's s
SLE: systemic lupus erythematosus
PMR: polymyalgia rheumatic
EULAR: European League Against Rheumatism
ANCA: antinuclear cytoplasmic antibody
ACPA: anti-cyclic citrullinated peptide
