# Supplementary figures and images for: Uncovering the flip side of immune checkpoint inhibitors: a comprehensive review of immune-related adverse events and predictive biomarkers
Source: Int J Biol Sci. 2024 Jan 1;20(2):621–42. doi: 10.7150/ijbs.89376 (PMC10758091; doi:10.7150/ijbs.89376)

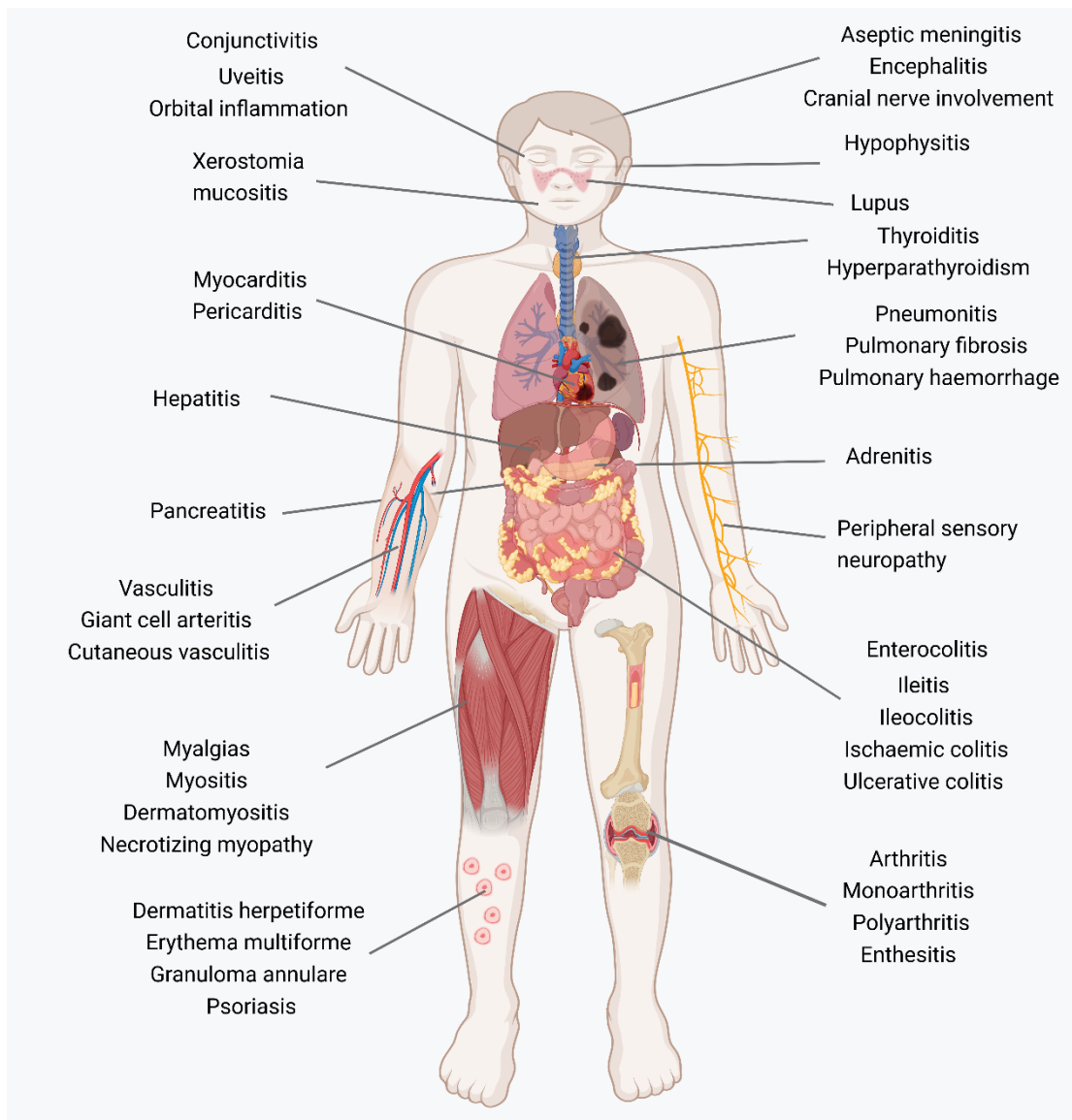

**Figure S1. Common immune-related adverse events in different organs.**

Supplement: Supplementary file 1 — Supplementary figure. [file ijbsv20p0621s1.pdf]
